# Acupucture in the treatment of scoliosis – a single blind controlled pilot study

**DOI:** 10.1186/1748-7161-3-4

**Published:** 2008-01-28

**Authors:** Hans-Rudolf Weiss, Silvia Bohr, Anja Jahnke, Sandra Pleines

**Affiliations:** 1 Asklepios Katharina Schroth Spinal Deformities Rehabilitation Centre, Korczakstrasse 2, D-55566 Bad Sobernheim, Germany

## Abstract

**Background:**

Today, acupuncture therapy is commonly used for pain control throughout the world, although the putative mechanisms are still unclear. A Pub Med search for the key words "Acupuncture" and "Scoliosis" reveals 3 papers only, not containing any results of studies designed for the treatment of scoliosis with the help of acupuncture. Because of this lack of trials especially designed for the treatment of scoliosis this pilot study has been performed.

**Methods:**

24 girls undergoing in-patient rehabilitation, 14 – 16 years of age (at average 15,1 years, SD 0,74) with the diagnosis of an Adolescent Idiopathic Scoliosis (AIS) have agreed to take part in this controlled single blind crossover study. Average Cobb angle was 33 degrees (SD 9,2) ranging from 16 to 49 degrees. 10 of the girls had a thoracic, one a lumbar, 7 a double major and 6 a thoracolumbar curve pattern. The patients have been scanned with the Formetric^® ^surface topography measurement system before and after lying on the left side [L], before and after sham acupuncture [S] and before and after real acupuncture [R].

**Results:**

For the whole group of patients no significant changes have been found during lying, sham acupuncture or real acupuncture. There were no differences between the patient groups with different curve pattern. In the explorative subgroup analysis of Patients with curvatures from 16 to 35 degrees, however significant changes in surface rotation have been found after R intervention as well as a strong differences in lateral deviation while in the L or S intervention no real changes have been achieved.

**Conclusion:**

One session with real (verum) acupuncture seems to have an influence on the deformity of scoliosis patients with no more than 35 degrees. The findings during verum acupuncture clearly are different to sham acupuncture or just lying, while in the whole group of patients also including patients with curvatures of more than 35 degrees no obvious changes have been found. The results of this study justify further investigation of the effect of acupuncture in the treatment of patients with scoliosis.

## Background

Acupuncture involves penetration of the skin by thin, solid, metallic needles that are stimulated either manually or electrically. It is one of several Traditional Chinese Medicine (TCM) therapies that have been used for thousands of years in the treatment of a variety of health problems. Today, acupuncture therapy is commonly used for pain control throughout the world, although the putative mechanisms still are unclear [1].

TCM is a complete system of healthcare delivery including Qi Gong [2], herbal medicine [3,4] and syndrome specific diets [4]. Therefore, a discussion of the efficacy of acupuncture must be prefaced with an understanding of the problems that occur when researchers attempt to evaluate treatments from one diagnostic paradigm (i.e., TCM) to diagnoses made with a different paradigm (i.e., allopathic medicine) [1]. Many studies have evaluated the use of acupuncture for symptoms that are either side effects of biomedical treatment (e.g., pain, fatigue) [1] or related to a specific disease process (e.g., OA). TCM syndromes, however, are not equivalent to either of these side effect types as defined by biomedical models. One must therefore use caution when interpreting results from studies that apply a TCM treatment to anything other than a TCM-related diagnosis or syndrome[1,3-5].

TCM syndromes, such as yin/yang deficiencies or qi stagnation, are unique symptom complexes that result from imbalances between the body's various functional systems. Since TCM is based on functional relationships and TCM diagnoses involve syndromes not diseases, no one-to-one correlation of signs and symptoms between TCM diagnoses and biomedical diseases exists. The diagnostic symptom complexes used in TCM differ from an allopathic model, and treatment is given according to individual patterns of system imbalances [1].

Hundreds of clinical trials evaluating the efficacy of acupuncture for various conditions have been conducted and recently RCT studies have been published on pain syndromes [1]. Many studies, however, were poorly designed and few included treatment protocols as applied in actual clinical practice. Nevertheless, in some areas related to pain management, reviews provide sufficient evidence of efficacy to draw clear conclusions [1].

Since the early 1970s, the efficacy of acupuncture for treating clinical conditions has been evaluated in several hundred randomized trials [6-10]. Results from these trials have been synthesized in systematic reviews. However considerable methodological diversity exists in the comprehensiveness of database searches for Cochrane systematic reviews on acupuncture. This diversity makes the reviews prone to bias and adds another layer [11].

Also the German GERAC committee has published various studies about the effect of acupuncture in disorders like low back pain, Osteoarthritis of the knee and Migraine [12-19].

We have used acupucture during Scoliosis In-patient Rehabilitation (SIR) regularly for 7 years now for the treatment of back pain and we were wondering as to whether acupuncture could also be helpful in treating the deformity itself.

A Pub Med search for the key words "Acupuncture" and "Scoliosis"reveals 3 papers [20-22] not containing results of studies especially designed for the treatment of scoliosis with the help of acupucture. Because of this lack of trials designed for the treatment of scoliosis this pilot study has been performed.

## Methods

### Material

24 girls undergoing in-patient rehabilitation, 14 – 16 years of age (at average 15,1 years, SD 0,74) with the diagnosis of an Adolescent Idiopathic Scoliosis (AIS) have agreed to take part in this study. Average Cobb angle was 33 degrees (SD 9,2) ranging from 16 to 49 degrees. 10 of the girls had a thoracic, one a lumbar, 7 a double major and 6 a thoracolumbar curve pattern. 17 girls wore a brace (part time during rehabilitation), 7 Patients had no brace.

The patients were instructed not to wear their braces three days prior to the start of the study.

### Methodology

9 Treatment couches have been provided and numbered. Because of practical reasons we decided to treat all patients in a comfortable position lying on the left side. In side lying position the first author was able to place needles front and back as well, in an easy way.

At first every patient had to be scanned with the Formetric^® ^surface topography measurement system. After that all patients had to lie down on the left side for 25 min. without any treatment. Thereafter the patients again have been scanned with the Formetric^® ^system in order to detect the effect of just lying. Then the patients have been divided into two groups. One group underwent real acupuncture on the first day of the study and the patients of this group were distributed to the equal couch numbers, the others received sham acupuncture and the patients of this group were distributed to the unequal couch numbers. After either treatment lasting 25 min. the patients have been scanned another time with the Formetric^® ^system. The patients did not know as to whether they received a real treatment or sham acupuncture.

The next day the groups have been changed to allow every patient to receive one session with real acupuncture and one session with sham acupuncture. Again, before treatment the patients have been scanned as well as after treatment.

For every patient after performance of the study we had one surface scan before just lying on the left side, after lying on the left side (25 min.), before sham acupuncture, after sham acupuncture (25 min.), before real acupuncture and after real acupuncture (25 min.) as well.

SB, AJ and SP have organized the patient distribution to the different couches and took the Formetric^® ^measurements.

### Acupucture points used

The first author having a more than 12 year experience in TCM has chosen the acupuncture points used in this pilot study.

**Figure 1:**
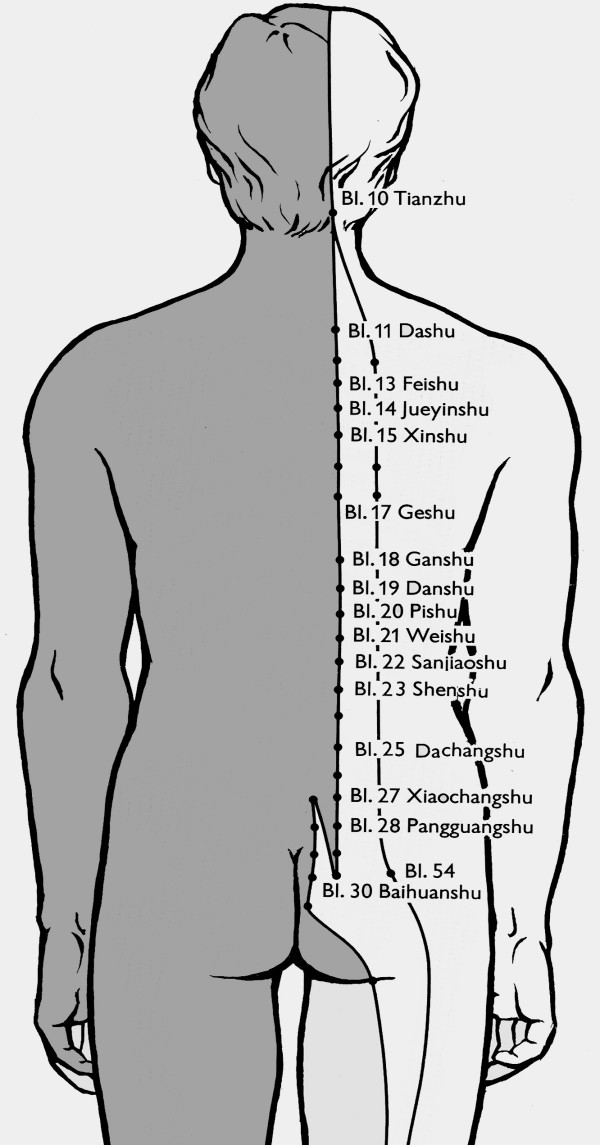
**Location of the Bladder meridian**. The bladder meridian has two branches on the back. Additionally to the basic concept of treatment the two acupuncture points on both branches of this meridian next to the deepest level of the concavity have been used [modified from 5].

In TCM the spine and back are closely related to the "Water Element" and the meridians of Kidney (KI) and Bladder (BL). Left and right of the spine the bladder meridian is located in a double layer (Fig. 1). The central channel (meridian) on the back is the Du Mai channel (DU). This is why these channels have to be addressed in first place in the treatment of scoliosis with acupuncture.

To speak in terms of Qi, Yin and Yang: Yin-Qi is related to material and coldness and Yang-Qi to function and heat.

If the channels around the spine have lost the power to keep it in line, we may assume a Yang-Qi deficiency. As a consequence the concept for acupuncture in the treatment of scoliosis should increase Yang-Qi in the channels named above.

The rational for the acupuncture point selection was:

1 Selection according to channels local distal points: UB 23, Du3, Du 4, Du 20, 67. SI 3 axis point.

According to syndromes: UB 67, KI 3

**Figure 2:**
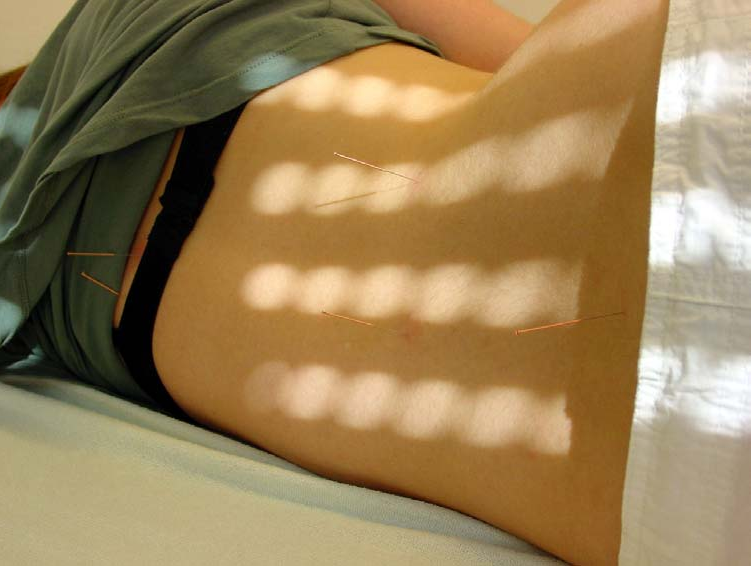
**Location of BL 23 Shenshu (normal needle)**. Location of the acupuncture point BL 23 Shenshu used on both sides. Point DU 3 Yaoyangguan (normal needle) is also visible on this picture on the right side. Additionally to that two points on the Bladder Meridian have been chosen in the concavity of the main curve, one on the central and one on the lateral part of the Bladder channel (normal needle on the left).

**Figure 3:**
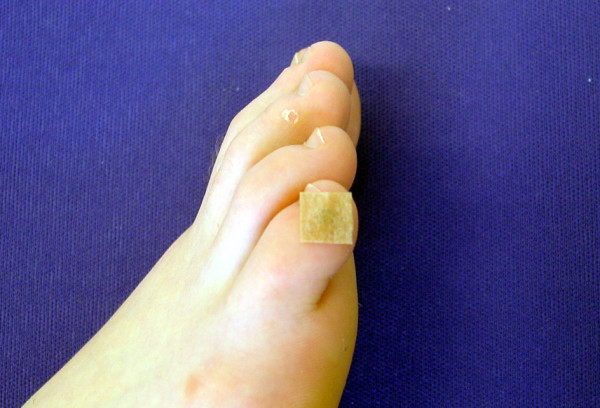
**Location of BL 67 Zhiyin right side (ear needle)**. Location of the acupuncture point BL 67 Zhiyin right side (ear needle).

**Figure 4:**
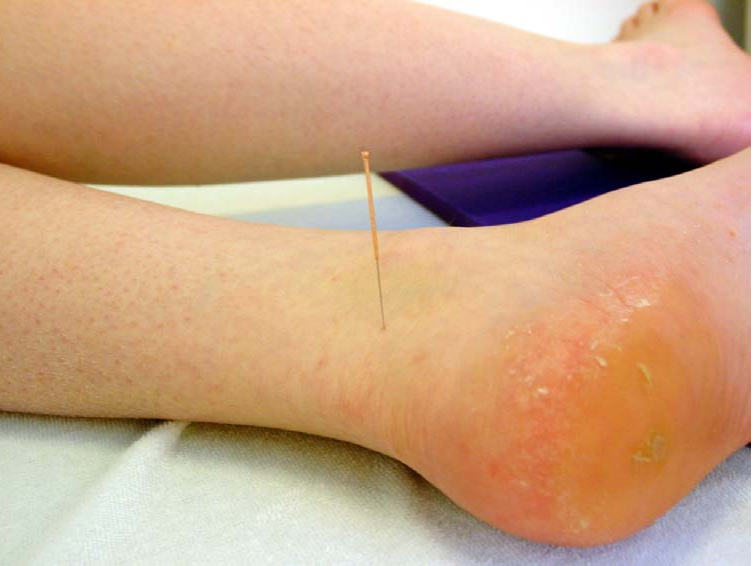
**Location of KI 3 Taixi left side (normal needle)**. Location of the acupuncture point KI 3 Taixi left side (normal needle).

**Figure 5:**
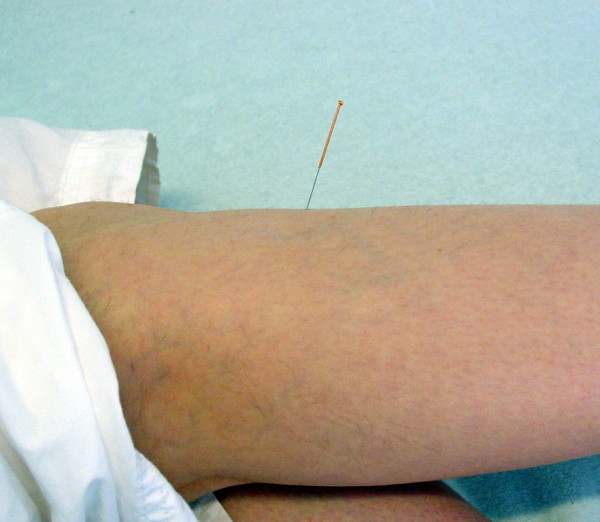
**Location of ST 36 Zusanli right side (normal needle)**. Location of the acupuncture point ST 36 Zusanli right side (Increase energy in Taixi; normal needle).

**Figure 6:**
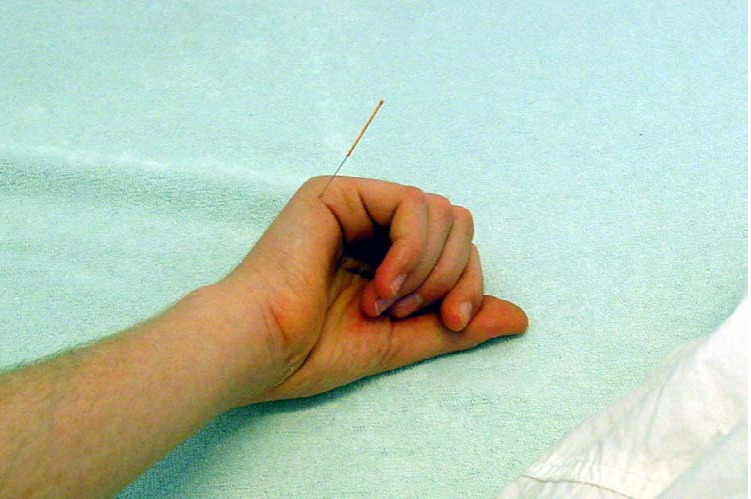
**Location of SI 3 Houxi (normal needle)**. Location of the acupuncture point SI 3 Houxi (Opens the Du Mai channel, normal needle).

General energetic points: ST 36, DU20 [5]:

The execution (type of needles, unilateral/bilateral) is described in the list of acupuncture points below:

1. BL 23 Shenshu both sides (normal needle, Fig. 2)

2. BL 67 Zhiyin right side (ear needle, Fig. 3)

3. KI 3 Taixi left side (normal needle, Fig. 4)

4. ST 36 Zusanli right side (Increase energy in Taixi; normal needle, Fig. 5)

5. DU 20 Baihui (normal needle)

6. DU 3 Yaoyangguan (normal needle)

7. SI 3 Houxi (Opens the Du Mai channel, normal needle, Fig. 6)

Additionally to that two points on the Bladder Meridian have been chosen in the concavity of the main curve, one on the central and one on the lateral part of the Bladder channel (normal needle).

The sham acupuncture consisted of points between two channels in the region of the regular points listed above with the same kind of needles as used in the real acupuncture, inserted the same way as the needles for real acupuncture. The patients did not recognize as to whether they were treated or not because they were not told about the possibility of receiving sham acupuncture before.

A possible "carry over" effect acupuncture/lying is largely ruled out by the fact that the patients were randomly assigned to the subgroups who had been lying on the left side [L] before sham acupuncture [S] or lying on the left side [L] before real acupuncture [R] on the first day of the study.

### Surface topography system used for this study

The print-out of the Formetric^®^-system itself gives a lot of values, the most important are the following: Lateral asymmetry, surface rotation and kyphotic angle.

**Figure 7:**
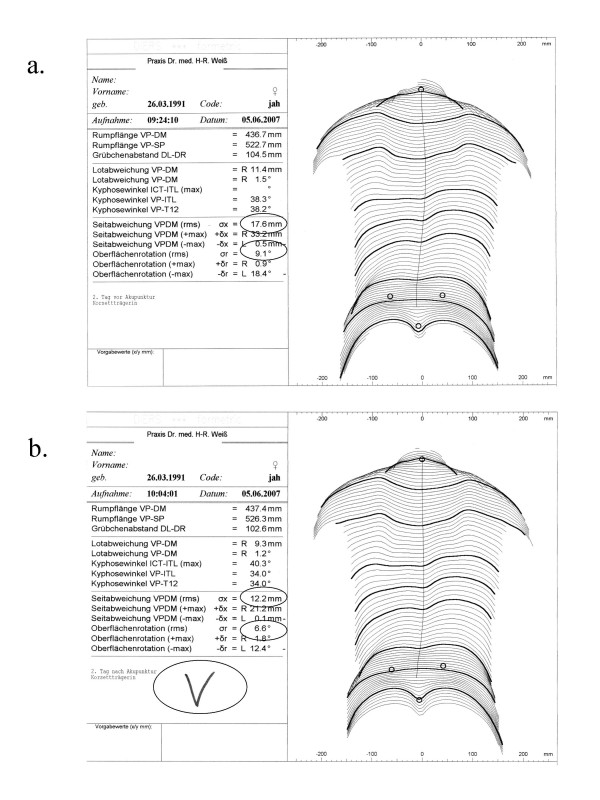
**Formetric^® ^scan before (a) and after (b) real (verum) acupuncture**. In the Formetric^® ^scan before (a) and after (b) real (verum) acupuncture an improvement of lateral deviation (Seitabweichung VPDM []rms]) as well as surface rotation (Oberflächenrotation [rms]) is visible.

In the video rasterstereography (Formetric^®^-system) the whole object is illuminated by a pattern of parallel lines, recorded in a single frame and needing only a short measurement time (40 msec). The automatic image processing consists of the identification of the raster lines and automatic 3-D reconstruction of the back; shape analysis is performed by a computer. The computer helps to evaluate the spine with the assistance of triangulation [23,24]. The video rasterstereography (Formetric^®^-system) has a point discrimination of 0.15 mm, and in a typical case of measurement 25000 surface points are calculated (Fig. 7).

Parameters used to compare the short-term effects of the different treatment concepts described were average lateral deformation (root mean square [rms]) with a technical error of 3 mm and average surface rotation (rms) with a technical error of 1,5° as measured and calculated by the Formetric^® ^surface topography system [25-28].

The surface topography values for this study were compared as follows:

The values for lateral deviation and surface rotation (rms) after a session (lying [L], sham [S] and real acupuncture [R]) were compared with the value before the session.

The surface topography values were compared statistically using Winstat^® ^Software.

## Results

### Results of the whole group

In none of the sessions [L, S or R](lying, sham and real acupuncture) significant changes have been found. There were no differences between the individual sessions especially not between just lying on the left side and real acupuncture. The results can be seen in table 1.

**Table 1:** Results of the whole patient sample. In none of the sessions [L, S or R](lying, sham and real acupuncture) significant changes have been found.

n = 24	Average Difference	Standard Deviation	p
Surface Rotation [L]	0,09	1,1	0,72
Lateral Deviation [L]	0,98	2,6	0,11
Surface Rotation [S]	0,29	1,1	0,21
Lateral Deviation [S]	0,19	2,8	0,73
Surface Rotation [R]	0,23	1,7	0,35
Lateral Deviation [R]	0,92	2,8	0,13

No differences have been found in the patient groups with different curve pattern. Brace treatment prior to the onset of the study also did not seem to make a difference.

However curve size seemed to make a difference and this is why we want to present the results of the subgroup of patients with no more than 35 degrees below.

### Results of the explorative subgroup analysis with curves from 16 to 35 degrees

Average Cobb angle in this group (n = 15) was 27 degrees (SD 6,1), average age 15,3 years (SD 0,7)

**Figure 8:**
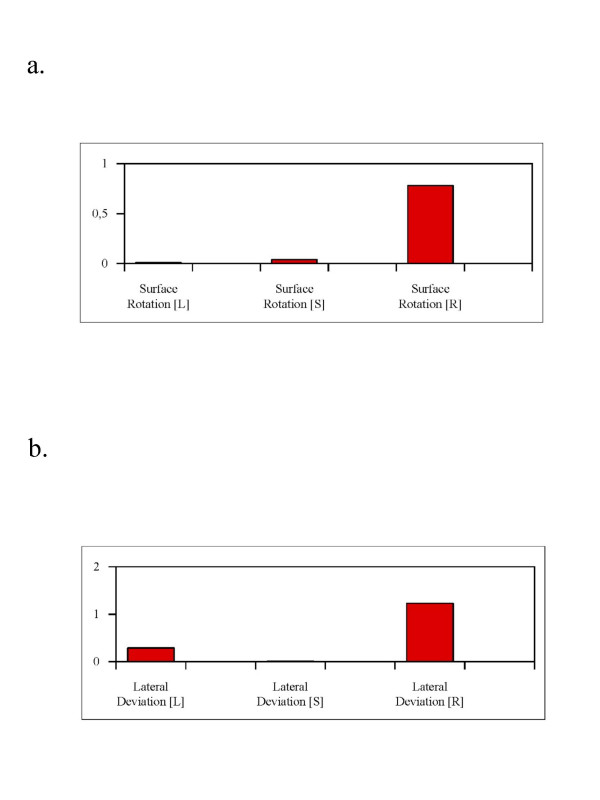
**Results of the patient sample with no more than 35°**. Figure 8a shows the results of surface rotation. The improvement (decrease) was highest after real (verum) acupuncture and statistically significant (see also table 2.). Figure 8b shows the results of lateral deviation. The improvement (decrease) was highest after real (verum) acupuncture, however statistically not significant (see also table 2.).

In the L and S session no big changes have been found, nor did we find  significant differences. In the R session however surface rotation improved  significantly and lateral deviation nearly significantly. The results of  this subgroup can be seen in table 2 and figure 8.

**Table 2:** Results of the patient sample with no more than 35°. The improvement of surface rotation (decrease) was highest after real (verum) acupuncture and statistically significant. The improvement of lateral deviation (decrease) was highest after real (verum) acupuncture, however statistically not significant.

n = 15	Average Difference	Standard Deviation	p
Surface Rotation [L]	0,01	1,1	0,96
Lateral Deviation [L]	0,29	2,6	0,67
Surface Rotation [S]	0,04	0,9	0,87
Lateral Deviation [S]	0,01	2,2	0,99
Surface Rotation [R]	0,78	1,3	0,04
Lateral Deviation [R]	1,23	3,1	0,13

## Discussion

**Figure 9:**
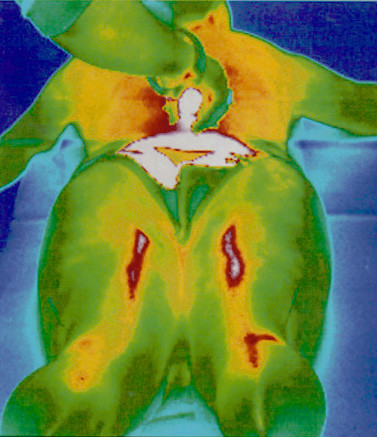
**Thermographical representation of the Bladder meridian**. Heat (Moxa) induced thermographical representation of the Bladder meridian. The lightened Moxa cigar is placed between the branches of the Bladder meridian at the level of BL 23/Du 4. The longitudinal spreading of the heat along a meridian-like pathway (Bladder meridian) is clearly visible (modified from [28]).

**Figure 10:**
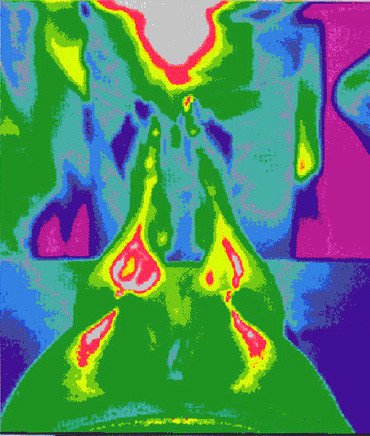
**Thermographical representation of the Spleen meridian**. Heat (Moxa) induced thermographical representation of the Spleen meridian. The lightened Moxa cigar is placed between the big toes where the point Spleen 1 is located on the medial side. The longitudinal spreading of the heat along a meridian-like pathway (Spleen meridian) is clearly visible (modified from [28]).

Although the mechanisms of TCM are still unclear, the concept of the "energy channels" or meridians has been shown existent. The application of heat on certain acupuncture points did not lead to a spherical spreading of the heat around the spot, but to a longitudinal spreading along meridian-like pathways (Fig. 9 and 10) [29]. These findings speak for the existence of meridian like channels. However, in the scientific acupuncture community the existence of channels is not regarded as proven. This paper [29] is discussed heavily and has not been published in a Medline listed journal and has not been replicated.

In literature we can additionally find strong evidence that  acupuncture may influence quite a number of pain syndromes [6-19] and  therefore acupuncture has been applied for long also at this centre in  patients with spinal deformities and pain [20]. Until now no acupuncture concept of treatment has been worked out for the treatment of the deformity itself. Having found some effect of acupuncture (significant improvement of surface rotation) in patients with curvatures on no more than 35 degrees in only one session has been surprising. In bigger patient samples significant results would have been expected for lateral deviation as well.

This finding has encouraged us to start a controlled study with scoliosis patients receiving a series of 6 acupuncture sessions during Scoliosis In-patient Rehabilitation (SIR).

Of course we have to take into account the technical error of the Formetric^®^-system which is bigger than the average changes after the individual sessions. However we have differences that are at least in part statistically significant comparing the different interventions (L, S and R) and also medically relevant differences justifying preliminary conclusions. We have of course to accept that there are limitations of this study due to the relatively big technical error of the measuring system (15 – 20% for Lateral Deviation and Surface Rotation; 5% for Kyphosis Angle [25-28]), however postural sway cannot be regarded as a systematic error and therefore we would expect small changes not to be registered with the help of this device whereas clear tendencies or significant changes erase from the general variability and so are medically relevant. On the other hand there is no other device to measure the effects of exercises in short-term objectively other than x-rays not applicable for a study of this design because of the exposition to radiation.

When postural sway is not regarded as a systematic error all significant changes may be interpreted as being of medical relevance, while in the individual case the changes cannot be interpreted just by the values. The picture printed out on the Formetric^®^-sheet (Fig. 7) therefore gives additional information, which however is also subject to postural sway.

There was a slight improvement of lateral deviation in the side lying intervention without any influence on surface rotation. This might be due to the fact that most of the curves were right thoracic and lying on the concavity unloads the curve without an influence on the deformity in 3D.

It has to be discussed why the changes seem relevant in the subgroup of patients with curvature of no more than 35 degrees while in the whole group of patients no relevant changes have been detected. As we know from certain brace studies bigger curves can be corrected not as easily as smaller curves [30,31]. The reason therefore might be stiffness increasing with curve size and age [30,31]. Therefore it might be possible that also patients with curvatures bigger than 35 degrees profit from a series of acupuncture.

In the very end it has to be discussed, as pointed out by Bunnel [32], that it has become apparent from many reports that, although there is a significant correlation between clinical deformity and radiographic measurement, the standard deviation is so high that it is not possible to reliably predict the degree of curvature from surface topography in any given patient by any technique" Bunnel [32] also states that, in general, clinical deformity is disproportionately greater than expected for the degree of Cobb angle in the early stages of the development of scoliosis.

## Conclusion

One session with real (verum) acupuncture seems to have an influence on the deformity of scoliosis patients with no more than 35 degrees. The findings during verum acupuncture clearly are different to sham acupuncture or just lying, while in the whole group of patients also including patients with curvatures of more than 35 degrees no obvious changes have been found. The results of this study justify further investigation of the effect of acupuncture in the treatment of patients with scoliosis.

## Competing interests

The author(s) declare that they have no competing interests.

## Authors' contributions

HRW: Study design, performance of treatments, manuscript writing.

SB, AJ and SP: Patient acquisition, patient grouping, organization.

All authors read and approved the final manuscript
